# Changes in retinal nerve fiber layer and vessel densities after scleral buckling in patients with rhegmatogenous retinal detachment observed by OCTA

**DOI:** 10.3389/fmed.2024.1492828

**Published:** 2024-12-13

**Authors:** Cuiwen Zhang, Linlin Liu, Yiping Jiang

**Affiliations:** ^1^The First Clinical Medical College of Gannan Medical University, Ganzhou, China; ^2^First Affiliated Hospital of Gannan Medical University, Ganzhou, China

**Keywords:** rhegmatogenous retinal detachment, scleral buckling, peripapillary retinal nerve fiber layer, peripapillary vessel densities, OCTA

## Abstract

**Purpose:**

To observe the changes in peripapillary retinal nerve fiber layer (RNFL) thickness and peripapillary vessel densities (VD) in patients with rhegmatogenous retinal detachment (RRD) after scleral buckling (SB) by OCTA.

**Methods:**

A total of 40 patients (40 eyes) with monocular RRD who underwent SB were included in the study, with the operated eyes (40 eyes) as the study group and the contralateral healthy eyes (40 eyes) as the control to analyse the changes in peripapillary RNFL thickness and VD before and after surgery. Data were analysed by paired samples *t*-test or Wilcoxon signed rank sum test.

**Results:**

Comparison of the peripapillary RNFL thickness in the 8 areas between the two groups during the 6-month follow-up period: All 8 peripapillary areas of the optic disc were statistically different before surgery, except for the tempo superior and tempo inferior, which were statistically different at each postoperative follow-up point, and the remaining 6 areas in the operated eyes group were progressively closer to those in the healthy eyes group, and there was no significant difference between the two groups. Comparison of peripapillary VD in the 8 areas between the two groups during the 6-month follow-up: Peripapillary VD in the 8 areas in the two groups were all statistically different before surgery, and except for superior tempo, which was statistically different at each postoperative follow-up time point, the remaining seven areas in the operated eyes group became progressively closer to that in the healthy eyes group and there was no significant difference.

**Conclusion:**

RRD negatively affects the peripapillary RNFL, but both peripapillary RNFL thickness and VD gradually improved in the operated eyes close to the contralateral eyes after SB.

## 1 Introduction

Rhegmatogenous retinal detachment (RRD) is a separation of the retinal nerve fiber layer from the pigment epithelial layer caused by the retinal hole. It is the most common type of retinal detachment in clinical practice, with an incidence of approximately 1 in 10,000 per year; and a blindness rate of nearly 100% if left untreated (1, 2). Scleral buckling (SB) and vitrectomy are the mainstay of treatment for RRD (3). Vitrectomy for RRD requires the injection of silicone oil or gas into the vitreous cavity (3, 4). Silicone oil is a commonly used intraocular filler because of its proximity to the vitreous. Silicone oil has been shown to hurt the optic nerve and retinal microcirculation (5). In contrast, SB for RRD consists of placing silicone strips or silicone sponges on the sclera corresponding to the fissure to create a depression in the sclera and choroid, reduce vitreous traction around the fissure, and seal the fissure. The effect on the optic nerve and retinal microcirculation around the optic disc has not been studied in detail.

Although visual acuity has been a commonly used clinical method to assess postoperative visual function, it is somewhat subjective, often macular dependent and does not provide a comprehensive, objective and accurate assessment of visual function (6). The ganglion cell body is located in the ganglion cell layer and its axons travel inward and parallel to the inner surface of the retina before entering the optic nerve to form the retinal nerve fibre layer (RNFL). RNFL thickness, which depends primarily on the number of ganglion cell axons, is an important indicator of quantitative response to changes in optic nerve and visual conduction function (7), which is directly related to visual acuity, contrast sensitivity, colour vision, visual field and other visual functions (8). The peripapillary vessel densities (VD) form a unique vascular network within the RNFL around the optic nerve head and are the innermost capillaries of the RNFL. They provide nutrients to the RNFL, which is relatively thicker in normal eyes in areas with higher VD and less spaced capillaries, and therefore thickest around the optic disc (9). In cotton-wool spots, intraretinal haemorrhages and ischaemic optic neuropathy, RNFL defects have an important correlation with the peripapillary VD, which has not been given enough attention in the past due to the difficulty of imaging it with certainty, but monitoring the peripapillary RNFL and VD is important for assessing visual function.

Evaluation of the tissues surrounding the optic disc provides information necessary for the diagnosis and evaluation of a wide range of ocular and neurological disorders (10). Optical coherence tomography angiography (OCTA) is a rapid and non-invasive ophthalmic imaging technique with the advantages of high resolution and reproducibility (11, 12); that can automatically measure retinal RNFL thickness and VD parameters in 8 regions around the optic disc. In this study, we investigated the changes in RNFL and VD after SB in patients with RRD using OCTA.

## 2 Materials and methods

### 2.1 General information

Patients who underwent SB for monocular RRD in our hospital between November 2021 and March 2023 were included in the study. The operated eyes was the study group and the contralateral healthy eyes was the control. Inclusion criteria: (1) Patients with a clinical diagnosis of monocular RRD whose contralateral eyes were normal (normal eyes had normal visual acuity, intraocular pressure, slit lamp, fundus and other examinations and had not received any treatment); (2) Refractive error between the RRD eye and the contralateral healthy eye of <3.00D; (3) Patients with RRD according to the American Retina Society’s 1983 PVR grading criteria (13): PVR grades A, B, and C; (4) no previous ocular surgical treatment; and (5) at least 6 months of follow-up. Exclusion criteria: (1) Patients with ocular and systemic diseases other than RRD, such as glaucoma, diabetes mellitus, hypertension, etc., (2) Patients with failed SB or recurrence of retinal detachment during the follow-up period; (3) Patients with macular lentigines; (4) Patients with pathology in the contralateral eye during the follow-up period; (5) Patients who cannot be clearly imaged on OCTA due to refractive interchromatic opacities, or whose 8 divisions of the peripapillary area cannot be measured on OCTA for various reasons; (6) Patients who cannot cooperate with the examination for various reasons; (7) Patients whose OCTA cannot be clearly imaged due to refractive interstitial opacities or whose data in all 8 peripapillary disc locations cannot be measured for various reasons; (8) Patients who cannot cooperate with the examination for various reasons. All patients underwent a comprehensive ophthalmic examination of both eyes at enrolment, including best corrected visual acuity, intraocular pressure, slit-lamp examination, fundus photography, indirect ophthalmoscopy, and OCTA. The study adhered to the tenets of the Declaration of Helsinki and was approved by the Ethics Committee of First Affiliated Hospital of Gannan Medical University. Informed consent was obtained prior to the inclusion of subjects.

### 2.2 Surgical techniques

Patients were fully informed of the possible risks and complications of SB before surgery, and patients or their relatives signed the informed consent form and then underwent surgery. Patients were placed in the recumbent position, anaesthetised under retrobulbar anaesthesia, the eyelids were opened with a lid opener, the bulbar conjunctiva was incised in a circular pattern according to the location of the retinal hole, the rectus muscle traction suture was fixed, and a 360-degree peripheral funduscopy was performed, and a 360-degree peripheral fundus examination was performed with a bimanual indirect ophthalmoscope under the upper pressure of the cryoprobe to find the retinal hole and the area of degeneration, and the retina around the hole and the area of degeneration was frozen, and condensation was stopped when the retinal pigment epithelium or the retina became whitish, and the sclera was located outside the hole and pre-positioned under the suture. The pre-positioned sutures were placed under the sutures with a silicone sponge and the pre-positioned sutures were tightened so that the fissure was well closed at the pressure ridge.

### 2.3 OCTA imaging

**Figure 1:**
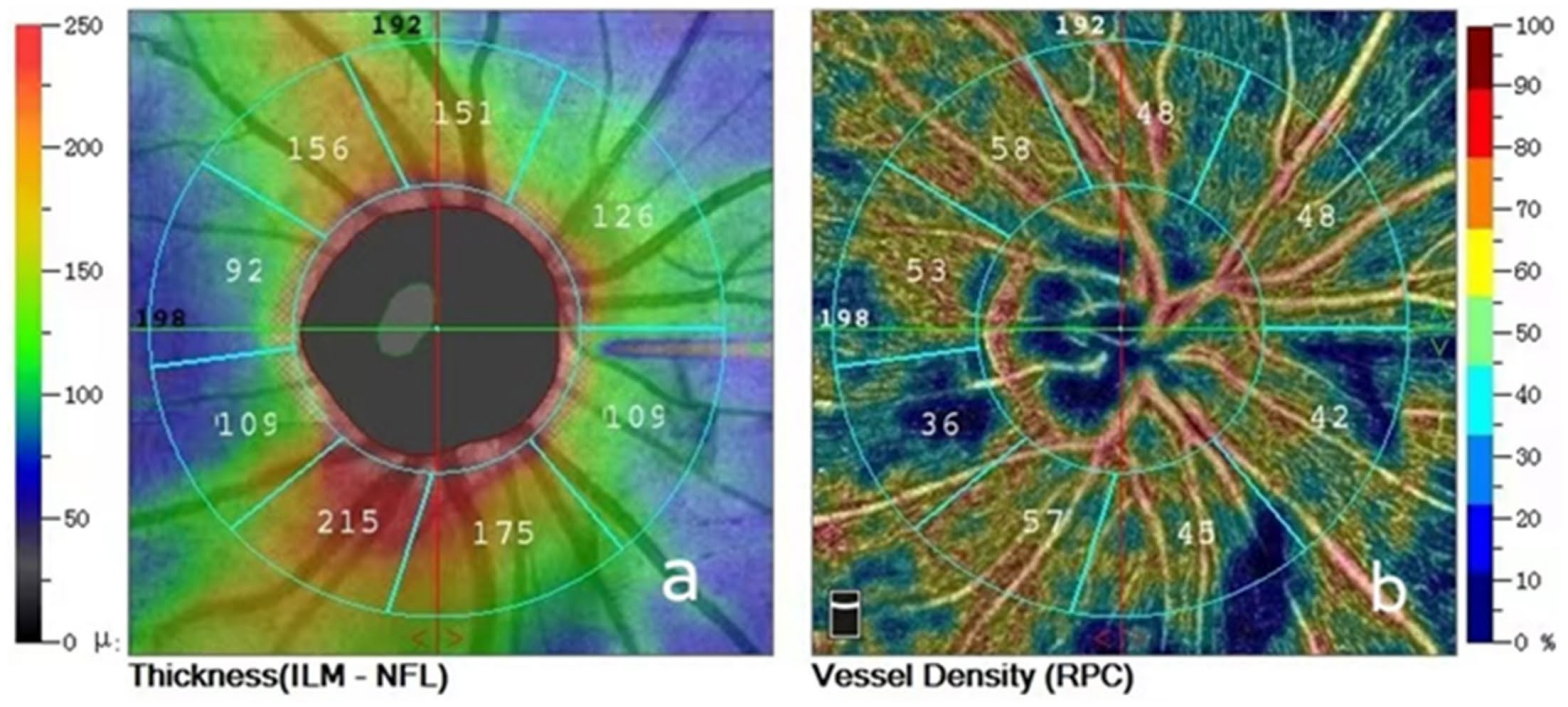
**(A)** Peripapillary retinal nerve fiber layer thickness divided into eight areas; **(B)** Peripapillary vessel densities divided into eight areas.

All patients were examined by trained and experienced clinical technologists. RNFL and VD images around the optic disc were obtained in both eyes using OCTA (Optovue RTVue XR Avanti, Optovue Inc., United States) and OCTA data were measured multiple times at each follow-up visit and the better quality images were selected for analysis. The Angio-Disc scan mode was selected for a 4.5 × 4.5 mm rectangular scan centred on the optic disc, the image was automatically segmented into a 2.0 mm diameter circle centred on the optic disc, and a circle 2.0 mm around this circle was defined as the peripapillary region, and the instrument delineated the peripapillary region as nasal superior, superior nasal, superior tempo, tempo superior, tempo inferior, inferior tempo, inferior nasal, nasal inferior. The instrument automatically detects RNFL and VD parameters in the above eight regions around the optic disc. The instrument automatically divides the optic disc periphery globally into 8 zones to generate RNFL and VD parameters (Figure 1) and automatically displays the signal strength index (SSI). In patients with RRD who underwent SB, intraocular pressure in both eyes, interocular ophthalmoscopy after pupil dilation, and OCTA were performed preoperatively and at 1 day, 2 weeks, 1 month, 3 months, and 6 months postoperatively to record RNFL thickness, VD in each quadrant around the optic disc and SSI in the operated and healthy eyes at different times.

### 2.4 Statistical methods

SPSS23.0 software was used for statistical analysis, and the measurement data conforming to normal distribution were expressed as the mean ± standard deviation (SD), and the comparison between the operated eyes group and the healthy eyes group was performed by the paired samples t-test; the non-normally distributed data were expressed as median [interquartile range], and the comparison between the operated eyes group and the healthy eyes group was performed by the *Wilcoxon* signed rank-sum test. Differences were considered statistically significant at *p* < 0.05.

## 3 Results

### 3.1 Basic data

Sixty-five patients were initially identified as subjects, of which 18 patients were lost to follow-up, 3 patients had recurrent RRD during follow-up, 4 patients also had RRD in the contralateral eyes during the 6-month follow-up period, and 40 patients (40 eyes) were finally included in the analysis, including 21 male and 19 female patients, with a mean age of (49.26 ± 15.25) years and a mean duration of RRD of (1.01 ± 1.31) months. The RRD-affected eyes (40 eyes) that underwent SB were the study group (operated eyes group) and the contralateral healthy eyes of the patients (40 eyes) were the control group (Table 1). The intraocular pressure of these 40 patients was within the normal range during the follow-up period.

**Table 1:** Patient information.

Parameter	Value
Age	49.26 ± 15.25
Time of retinal detachment	1.01 ± 1.31
Gender	
Male	21 (52.5%)
Female	19 (47.5%)
Range of retinal detachment	
1 quadrant	12 (30.0%)
2 quadrant	18 (45.0%)
3 quadrant	10 (25.0%)
Surgical pressure range	
1 quadrant	18 (45.0%)
2 quadrant	22 (55.0%)

### 3.2 Comparison of SSI between the operated and healthy eye groups at different time points

The SSI was smaller in the operated eye group than in the healthy eye group at 1 day (6.600 ± 0.87 vs. 8.325 ± 0.92; *p* < 0.001) and 2 weeks (8.125 ± 0.82 vs. 8.550 ± 0.75; *p* = 0.022) after surgery, and the SSI was lower in the operated eye group than in the healthy eye group at 1 day preoperatively (8.025 ± 0.73 vs. 8.250 ± 1.17; *p* = 0.246), 1 month postoperatively (8.150 ± 0.86 vs. 8.300 ± 0.99; *p* = 0.430), 3 months postoperatively (8.175 ± 1.04 vs. 8.250 ± 1.06 *p* = 0.701), and 6 months postoperatively (8.125 ± 1.02 vs. 8.150 ± 1.05; *p* = 0.905) the operated eye group was not significantly different from the healthy eye group (Table 2).

**Table 2:** Comparison of preoperative and postoperative SSI at different time points between the operated eye group and the healthy eye group.

SSI	Operated eye group	Healthy eye group	*t*	*p*
Preoperative	8.070 ± 0.74	8.279 ± 1.14	−1.177	0.246
1 day postoperatively	6.767 ± 1.04	8.372 ± 0.90	−8.376	<0.001
2 weeks postoperatively	8.186 ± 0.82	8.581 ± 0.73	−2.369	0.022
1 month postoperatively	8.209 ± 0.86	8.348 ± 0.97	−0.798	0.429
3 month postoperatively	8.233 ± 1.02	8.302 ± 1.04	−0.387	0.701
6 month postoperatively	8.186 ± 1.01	8.209 ± 1.04	−0.121	0.904

#### 3.2.1 Comparison of peripapillary RNFL between the operated eye and the healthy eye groups at different preoperative and postoperative time points of the SB

At 6-month follow-up, the results of the corresponding comparison of peripapillary RNFL thickness between the operated eye group and the healthy eye group, respectively, showed that, preoperatively, the operated eye group had nasal superior (111 [96–134] vs. 105 [94–116]; *p* = 0.014), superior nasal (156 [120–191] vs. 138 [114–155]; *p* = 0.009), superior tempo (149 [126–200] vs. 133 [122–149]; *p* = 0.003), tempo superior (99 [81–211] vs. 86 [78–95]; *p* < 0.001), tempo inferior (85 [74–135] vs. 76 [64–86]; *p* < 0.001), inferior tempo (165 [131–188] vs. 152 [135–165]; *p* = 0.046), inferior nasal (156 [137–182)] vs. 143 [129–155]; *p* = 0.003), nasal inferior (104 [89–128] vs. 86 [76–99]; *p* < 0.001), and global (126.75 [115.13–133.13] vs. 115.75 [108.88–122.88]; *p* < 0.001) had a greater RNFL than the healthy eye group prior to surgery; beginning at 1 month postoperatively, the superior tempo in both groups (143.00 [122.00–155.00] vs. 134.00 [123.00–146.00]; *p* = 0.05) and inferior nasal (148.00 [129.00–161.00] vs. 143.00 [127.00–154.00]; *p* = 0.491) were not significantly different; starting at 3 months postoperatively, the two groups had a higher superior nasal (145.00 [128.00–162.00] vs. 136.00 [115.00–155.00]; *p* = 0.069), superior tempo (141.00 [119.00–155.00] vs. 135.00 [120.00–148.00]; *p* = 0.118), inferior tempo (165.00 [139.00–179.00] vs. 156.00 [134.00–167.00]; *p* < 0.074), inferior nasal (143.00 [129.00–157.00] vs. 142.00 [129.00–154.00]; *p* = 0.711), nasal inferior (90.00 [82.00–107.00] vs. 86.00 [78.00–102.00]), *p* < 0.053) were not significantly different; at 6 months postoperatively, only tempo inferior (88.00 [75.00–99.00] vs. 79.00 [68.00–86.00]; *p* < 0.001 and global (124.29 [113.00–129.71] vs. 116.63 [109.75–124.25]; *p* < 0.001, the operated eye group was still larger than the healthy side group (Table 3).

**Table 3:** Comparison of peripapillary RNFL between the operated eye and the healthy eye at different preoperative and postoperative time points of the SB (μm).

	Preoperative		*Z*	*p*	1 day postoperatively		*Z*	*p*
	Operated eye group	Healthy eye group			Operated eye group	Healthy eye group		
NS	111.00 [96.00–134.00]	105.00 [94.00–116.00]	−2.47	0.014	113.00 [101.00–129.00]	104.00 [93.00–114.00]	−2.808	0.005
SN	156.00 [120.00–191.00]	138.00 [114.00–155.00]	−2.609	0.009	142.00 [133.00–171.00]	130.00 [110.00–150.00]	−3.089	0.002
ST	149.00 [126.00–200.00]	133.00 [122.00–149.00]	−3.008	0.003	132.00 [114.00–151.00]	135.00 [120.00–148.00]	−0.755	0.450
TS	99.00 [81.00–211.00]	86.00 [78.00–95.00]	−4.058	<0.001	88.00 [77.00–95.00]	89.00 [79.00–98.00]	−2.368	0.018
TI	85.00 [74.00–135.00]	76.00 [64.00–86.00]	−4.246	<0.001	86.00 [79.00–103.00]	78.00 [66.00–85.00]	−4.342	<0.001
IT	165.00 [131.00–188.00]	152.00 [135.00–165.00]	−1.999	0.046	162.00 [141.00–184.00]	155.00 [135.00–165.00]	−2.096	0.036
IN	156.00 [137.00–182.00]	143.00 [129.00–155.00]	−3.007	0.003	148.00 [126.00–163.00]	145.00 [126.00–155.00]	−0.163	0.870
NI	104.00 [89.00–128.00]	86.00 [76.00–99.00]	−4.052	<0.001	90.00 [80.00–102.00]	84.00 [75.00–95.00]	−2.048	0.041
Global	138.25 [125.25–151.63]	115.75 [108.38–124.25]	−5.687	<0.001	121.50 [112.50–136.25]	114.50 [108.00–123.00]	−3.792	<0.001

	2 weeks postoperatively		*Z*	*p*	1 month postoperatively		*Z*	*p*
	Operated eye group	Healthy eye group			Operated eye group	Healthy eye group		
NS	113.00 [103.00–124.00]	106.00 [97.00–117.00]	−3.246	0.001	113.00 [102.00–123.00]	106.00 [98.00–116.00]	−2.639	0.008
SN	113.00 [103.00–124.00]	106.00 [97.00–117.00]	−3.246	0.001	149.00 [130.00–164.00]	136.00 [114.00–155.00]	−2.178	0.029
ST	146.00 [123.00–161.00]	135.00 [124.00–149.00]	−1.951	0.051	143.00 [122.00–155.00]	134.00 [123.00–146.00]	−1.903	0.057
TS	93.00 [80.00–100.00]	85.00 [77.00–95.00]	−2.651	0.008	92.00 [80.00–100.00]	86.00 [77.00–95.00]	−2.815	0.005
TI	85.00 [81.00–100.00]	76.00 [65.00–83.00]	−4.789	<0.001	87.00 [78.00–100.00]	75.00 [65.00–85.00]	−4.428	<0.001
IT	173.00 [142.00–185.00]	155.00 [136.00–165.00]	−3.225	0.001	160.00 [138.00–180.00]	155.00 [135.00–165.00]	−2.279	0.023
IN	155.00 [131.00–168.00]	126.00 [126.00–156.00]	−1.999	0.046	148.00 [129.00–161.00]	143.00 [127.00–154.00]	−0.689	0.491
NI	90.00 [83.00–111.00]	85.00 [77.00–98.00]	−2.627	0.009	94.00 [82.00–109.00]	88.00 [78.00–100.00]	−2.537	0.011
Global	126.75 [115.13–133.13]	115.75 [108.88–122.88]	−4.927	<0.001	124.38 [113.63–130.38]	116.63 [108.25–123.75]	−4.352	<0.001

	3 month postoperatively		*Z*	*p*	6 month postoperatively		*Z*	*p*
	Operated eye group	Healthy eye group			Operated eye group	Healthy eye group		
NS	110.00 [102.00–119.00]	105.00 [98.00–113.00]	−2.038	0.042	108.00 [100.00–119.00]	106.00 [98.00–114.00]	−1.459	0.145
SN	145.00 [128.00–162.00]	136.00 [115.00–155.00]	−1.821	0.069	135.00 [116.00–160.00]	138.00 [115.00–154.00]	−0.984	0.325
ST	141.00 [119.00–155.00]	135.00 [120.00–148.00]	−1.563	0.118	143.00 [122.00–155.00]	134.00 [123.00–146.00]	−1.903	0.057
TS	93.00 [81.00–101.00]	85.00 [76.00–93.00]	−3.128	0.002	92.00-[80.00–100.00]	86.00 [77.00–95.00]	−2.815	0.005
TI	88.00 [79.00–100.00]	79.00 [66.00–85.00]	−4.772	<0.001	88.00 [75.00–99.00]	79.00 [68.00–86.00]	−4.321	<0.001
IT	165.00 [139.00–179.00]	156.00 [134.00–167.00]	−1.787	0.074	160.00 [134.00–175.00]	156.00 [132.00–168.00]	0.196	0.196
IN	143.00 [129.00–157.00]	142.00 [129.00–154.00]	−0.37	0.711	147.00 [129.00–157.00]	143.00 [130.00–156.00]	−0.25	0.803
NI	90.00 [82.00–107.00]	86.00 [78.00–102.00]	−1.938	0.053	89.00 [80.00–103.00]	86.00 [78.00–102.00]	−1.314	0.189
Global	120.63 [114.13–130.50]	116.00 [110.50–124.50]	−4.009	<0.001	124.29 [113.00–129.71]	116.63 [109.75–124.25]	−4.021	<0.001

#### 3.2.2 Comparison of global peripapillary RNFL in the operated eye group and the healthy eye group at different preoperative and postoperative time points of the SB

**Figure 2:**
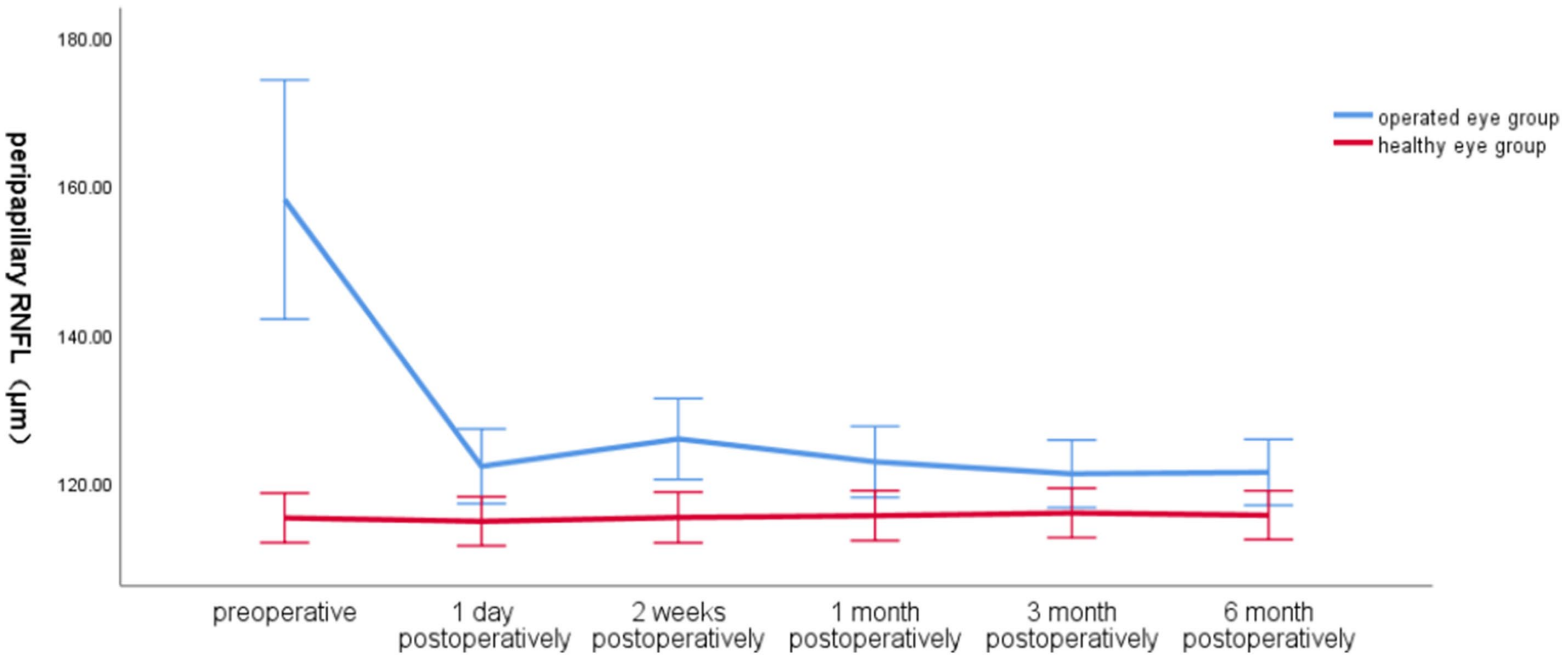
Line graph of global RNFL around the optic disc over time in the operated and healthy eye groups.

Preoperatively, the global RNFL around the optic disc in the operated eye group was significantly larger than that in the healthy eye group, the RNFL around the optic disc in the operated eye group decreased significantly on the first day of surgery, increased at 2 weeks postoperatively, and then gradually became smaller and closer to that of the healthy eye, with less fluctuation of the RNFL in the healthy eye group (Figure 2).

#### 3.2.3 Global RNFL and SSI over time around the optic disc in the operated eye group

**Figure 3:**
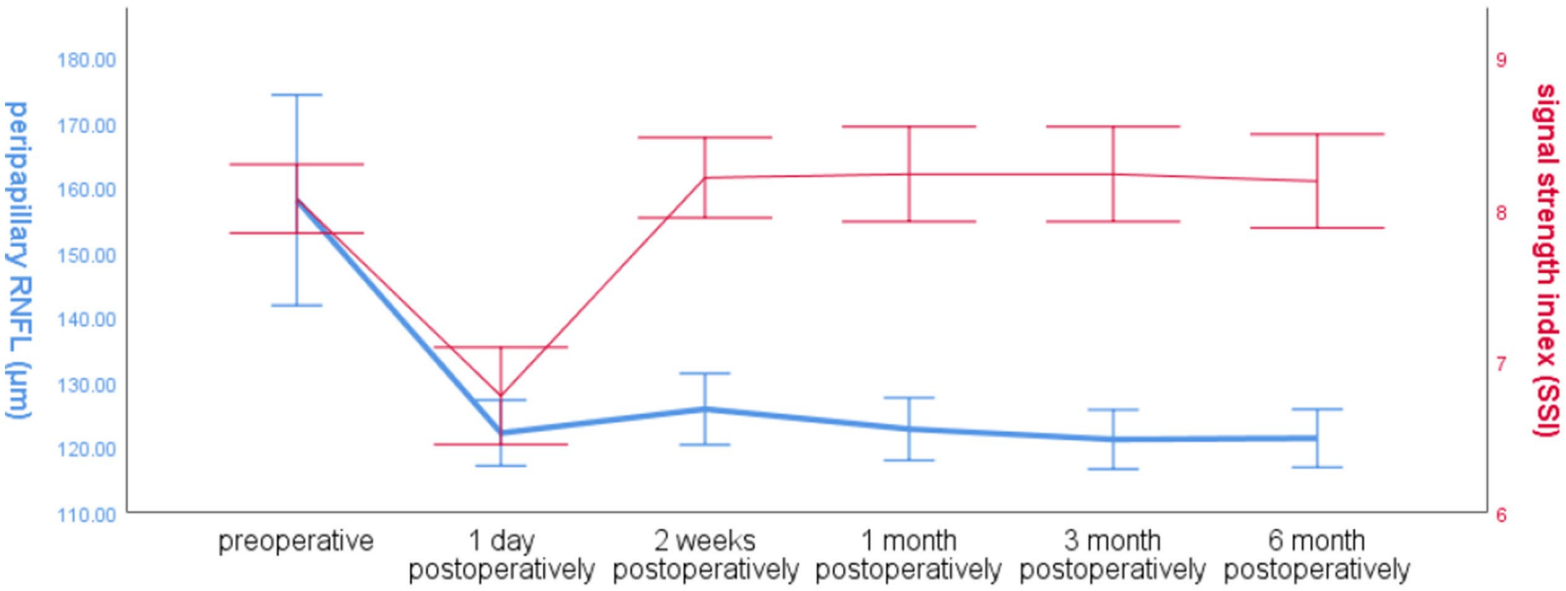
Line graph of global RNFL mean and SSI over time around the optic disc in the operated eye group.

The global RNFL around the optic disc in the operated eye group decreased significantly at 1 day postoperatively and increased slightly at 2 weeks postoperatively, and the trend of SSI changes in the operated eye group was the same as that of RNFL before 2 weeks postoperatively, and the SSI increased significantly at 2 weeks postoperatively, and was more fluctuating and less fluctuating at 2 weeks, and the trend of RNFL changes was different (Figure 3).

#### 3.2.4 Comparison of peripapillary VD between the operated eye and the healthy eye group at different preoperative and postoperative time points of the SB

Comparison of peripapillary VD between the operated and fellow eyes at 6-month follow-up showed that: preoperatively, the operated group had nasal superior (47.00 [43.00–48.00] vs. 49.00 [47.00–51.00]; *p* = 0.001), nasal superior (48.00 [43.00–52.00] vs. 52.00 [49.00–55.00]; *p* < 0.001), temporal superior (50.00 [44.00–55.00] vs. 56.00 [52.00–59.00]; *p* < 0.001), temporal superior (53.00 [47.00–57.00] vs. 56.00 [53.00–58.00], *p* = 0.016), inferior tempo (49.00 [44.00–52.00] vs. 53.00 [48.00–56.00]; *p* = 0.001), inferior tempo (55.00 [47.00–58.00] vs. 58.00 [53.00–60.00]; *p* = 0.004), inferior nasal (49.00 [45.00–53.00] vs. 53.00 [51.00–55.00]; *p* < 0.001), inferior nasal (46.00 [42.00–49.00] vs. 48.00 [45.00–50.00]; *p* = 0.027) and global (48.88 [38.88–45.88] vs. 53.00 [50.88–54.88]; *p* < 0.001) had smaller VD preoperatively than the healthy eye group; starting 1 month postoperatively, both nasal superior (48.00 [45.00–50.00] vs. 49.00 [47.00–50.00]; *p* = 0.063), tempo superior (55.00 [53.00–58.00] vs. 57.00 [54.00–59.00]; *p* = 0.516), tempo inferior (51.00 [48.00–54.00] vs. 53.00 [49.00–56.00]; *p* = 0.126) did not show significant differences; from 3 months postoperatively, the two groups nasal superior (50.00 [46.00–51.00] vs. 49.00 [46.00–51.00]; *p* = 0.907), superior nasal (51.00 [47.00–54.00] vs. 51.00 [48.00–57.00]; *p* = 0.504), tempo superior (57.00 [55.00–59.00] vs. 56.00 [54.00–59.00]; *p* = 0.346), tempo inferior (53.00 [49.00–56.00] vs. 54.00 [50.00–55.00]; *p* = 0.504), nasal inferior (48.00 [44.00–51.00] vs. 47.00 [44.00–50.00]; *p* = 0.434) were not significantly different; at 6 months only the superior temporal side (54.00 [51.00–56.00] vs. 55.00 [51.00–58.00]; *p* = 0.013) remained smaller in the operated eye group than in the healthy eye group (Table 4).

**Table 4:** Comparison of peripapillary VD between the operated eye and the healthy eye group at different preoperative and postoperative time points of the SB (%).

	Preoperative		*Z*	*p*	1 day postoperatively		*Z*	*p*
	Operated eye group	Healthy eye group			Operated eye group	Healthy eye group		
NS	47.00 [43.00–48.00]	49.00 [47.00–51.00]	−3.384	0.001	45.00 [40.00–49.00]	50.00 [47.00–52.00]	−4.051	<0.001
SN	48.00 [43.00–52.00]	52.00 [49.00–55.00]	−4.314	<0.001	48.00 [42.00–51.00]	52.00 [49.00–56.00]	−3.659	<0.001
ST	50.00 [44.00–55.00]	56.00 [52.00–59.00]	−4.23	<0.001	52.00 [47.00–54.00]	54.00 [51.00–59.00]	−3.429	0.001
TS	53.00 [47.00–57.00]	56.00 [53.00–58.00]	−2.408	0.016	52.00 [48.00–57.00]	57.00 [54.00–59.00]	−3.648	<0.001
TI	49.00 [44.00–52.00]	53.00 [48.00–56.00]	−3.289	0.001	50.00 [46.00–55.00]	52.00 [48.00–55.00]	−2.344	0.019
IT	55.00 [47.00–58.00]	58.00 [53.00–60.00]	−2.853	0.004	54.00 [48.00–57.00]	56.00 [55.00–60.00]	−4.64	<0.001
IN	49.00 [45.00–53.00]	53.00 [51.00–55.00]	−4.345	<0.001	48.00 [43.00–52.00]	52.00 [50.00–54.00]	−4.08	<0.001
NI	46.00 [42.00–49.00]	48.00 [45.00–50.00]	−2.214	0.027	42.00 [36.00–47.00]	48.00 [45.00–49.00]	−4.508	<0.001
Global	48.88 [38.88–45.88]	53.00 [50.88–54.88]	−5.712	<0.001	49.00 [46.00–50.38]	53.00 [50.75–54.75]	−5.12	<0.01

	2 weeks postoperatively		*Z*	*p*	1 month postoperatively		*Z*	*p*
	Operated eye group	Healthy eye group			Operated eye group	Healthy eye group		
NS	46.00 [43.00–49.00]	49.00 [46.00–51.00]	−3.648	<0.001	48.00 [45.00–50.00]	49.00 [47.00–50.00]	−1.856	0.063
SN	48.00 [45.00–53.00]	50.00 [47.00–55.00]	−2,712	0.007	49.00 [45.00–53.00]	51.00 [48.00–55.00]	−2.464	0.014
ST	52.00 [50.00–56.00]	55.00 [51.00–58.00]	−2.625	0.012	52.00 [50.00–56.00]	55.00 [51.00–58.00]	−2.292	0.022
TS	54.00 [52.00–57.00]	56.00 [54.00–59.00]	−3.01	0.003	55.00 [53.00–58.00]	57.00 [54.00–59.00]	−0.649	0.516
TI	50.00 [46.00–55.00]	52.00 [49.00–56.00]	−2.064	0.039	51.00 [48.00–54.00]	53.00 [49.00–56.00]	−1.529	0.126
IT	56.00 [50.00–59.00]	57.00 [54.00–60.00]	−2.439	0.015	55.00 [52.00–59.00]	56.00 [54.00–60.00]	−2.714	0.007
IN	48.00 [45.00–53.00]	53.00 [51.00–54.00]	−3.965	<0.001	50.00 [47.00–54.00]	52.00 [51.00–54.00]	−2.526	0.012
NI	44.00 [40.00–47.00]	46.00 [44.00–49.00]	−3.312	0.001	47.00 [42.00–48.00]	47.00 [44.00–49.00]	−2.319	0.020
Global	50.38 [48.13–51.75]	52.38 [51.13–54.38]	−4.704	<0.001	51.25 [48.00–52.88]	52.38 [51.00–53.88]	−3.163	0.002

	3 month postoperatively		*Z*	*p*	6 month postoperatively		*Z*	*p*
	Operated eye group	Healthy eye group			Operated eye group	Healthy eye group		
NS	50.00 [46.00–51.00]	49.00 [46.00–51.00]	−0.117	0.907	50.00 [47.00–52.00]	50.00 [46.00–51.00]	−1.337	0.181
SN	51.00 [47.00–54.00]	51.00 [48.00–57.00]	−0.668	0.504	51.00 [48.00–55.00]	51.00 [48.00–56.00]	−0.816	0.389
ST	53.00 [50.00–55.00]	55.00 [52.00–58.00]	−3.093	0.002	54.00 [51.00–56.00]	55.00 [51.00–58.00]	−2.491	0.013
TS	57.00 [55.00–59.00]	56.00 [54.00–59.00]	−0.942	0.346	56.00 [54.00–59.00]	56.00 [54.00–58.00]	−0.666	0.506
TI	53.00 [49.00–56.00]	54.00 [50.00–55.00]	−0.669	0.504	53.00 [48.00–56.00]	53.00 [49.00–56.00]	−1.5	0.134
IT	55.00 [53.00–58.00]	58.00 [55.00–60.00]	−2.428	0.015	57.00 [53.00–60.00]	58.00 [53.00–59.00]	−1.27	0.204
IN	50.00 [48.00–54.00]	52.00 [51.00–54.00]	−2.567	0.010	52.00 [49.00–55.00]	53.00 [51.00–55.00]	−1.351	0.177
NI	48.00 [44.00–51.00]	47.00 [44.00–50.00]	−0.783	0.434	48.00 [45.00–51.00]	46.00 [43.00–49.00]	−1.663	0.096
Global	52.38 [48.75–53.63]	53.25 [51.00–54.38]	−1.997	0.046	52.75 [49.88–54.38]	52.50 [51.00–54.25]	−0.075	0.940

#### 3.2.5 Changes in peripapillary global VD of the optic disc over time in the operated and healthy eye groups

**Figure 4:**
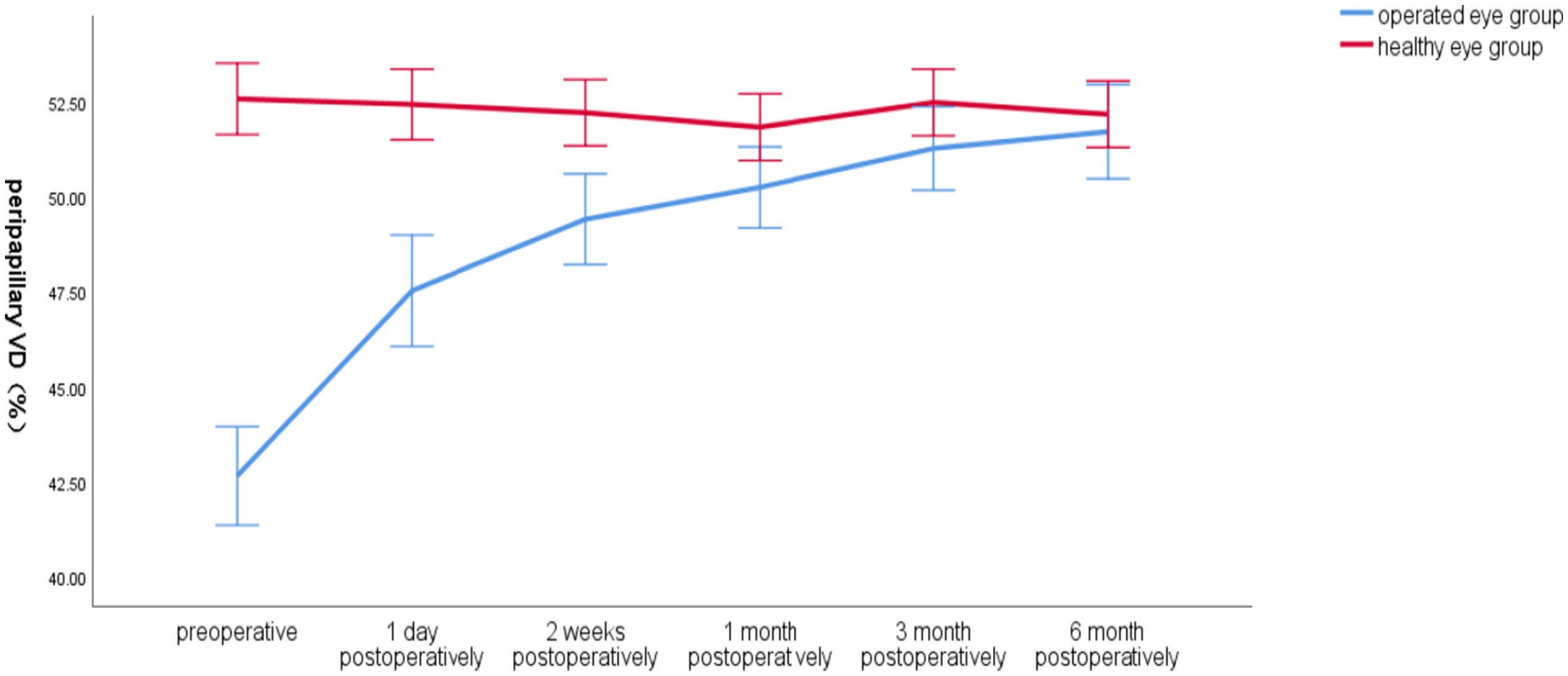
Line graph of mean global VD around the optic disc over time in the operated and healthy eye groups.

Preoperatively, the total VD around the optic disc in the operated eye group was significantly smaller than that in the healthy eye group, and postoperatively, the VD gradually increased closer to that in the healthy eye group, which had less fluctuation in VD (Figure 4).

#### 3.2.6 Global VD and SSI over time around the optic disc in the operated eye group

**Figure 5:**
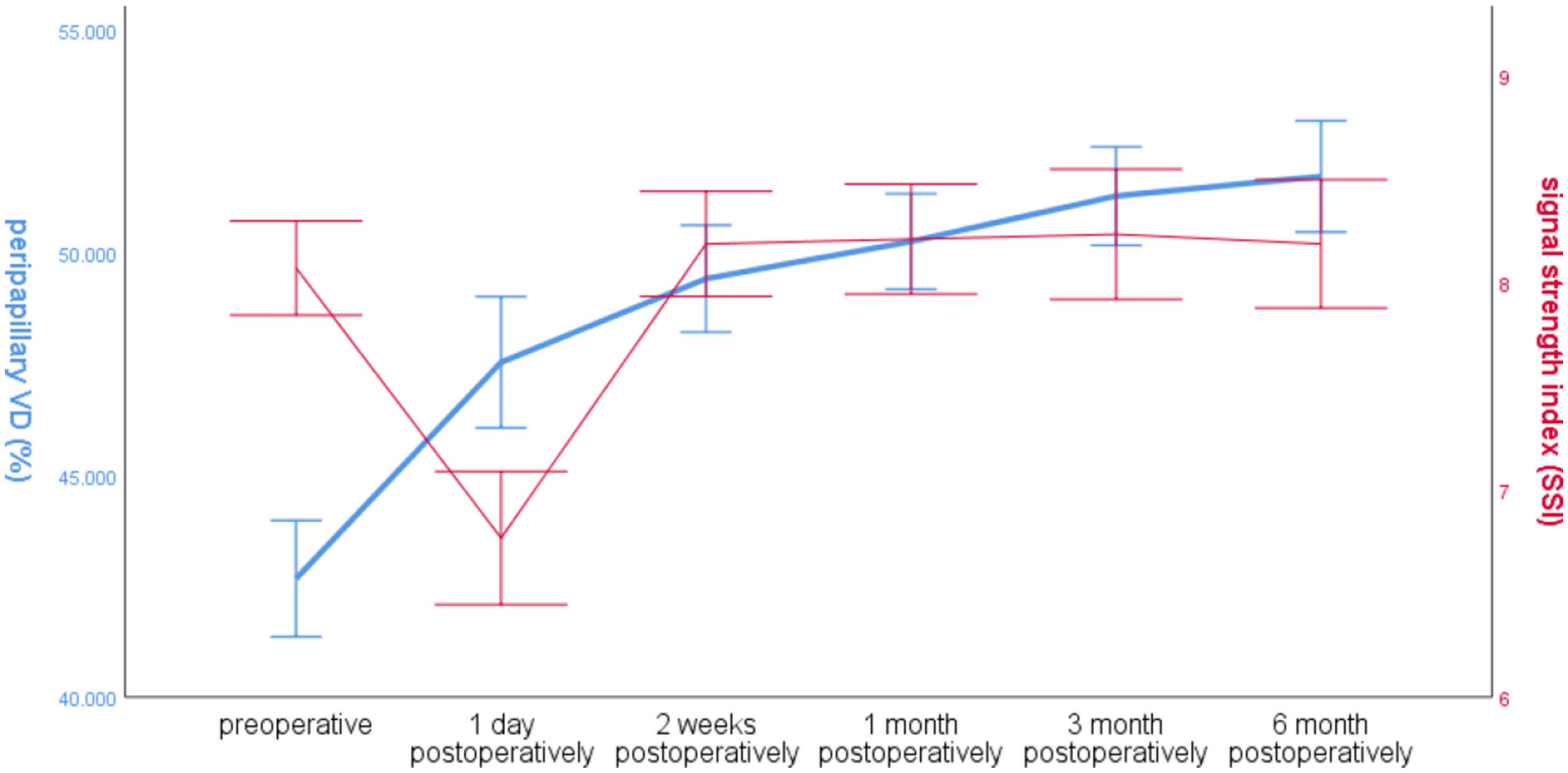
Line graph of global RNFL mean and SSI over time around the optic disc in the operated eye group.

The SSI in the operated eye group decreased significantly at 1 day postoperatively and increased significantly at 2 weeks postoperatively, and the overall VD around the optic disc increased. The trends of the two changes were different (Figure 5).

## 4 Discussion

In the early stages of RRD, patients have floating objects, flashing sensations or curtain-like occlusions in front of the eyes that gradually expand and severely affect vision, or even have only light sensation or no light sensation, and early diagnosis and treatment are crucial to save vision (14). SB and vitrectomy are currently accepted as the main treatment for RRD (3). Among them, vitrectomy for RRD requires the injection of fillers into the vitreous cavity, and silicone oil is one of the main fillers used for vitrectomy, which has been shown to have toxic effects on the retina (5, 15), resulting in significantly lower peripapillary VD than that of normal eyes (9), and a gradual decrease in RNFL thickness after surgery, especially in the retinal area over the optic disc. After silicone oil removal, peripapillary VD increased slightly but was still smaller than that of the contralateral normal eyes, while RNFL recovery was not evident (16–18). RNFL thickness and VD are closely related to visual acuity (19, 20). RNFL thickness is mainly determined by the number of ganglion cell axons and is an important index to quantitatively respond to changes in optic nerve cell function and visual conduction (7); VD indicates the supply of nutrients to the retina (9). Insufficient blood supply affects retinal cell function (21). This is why it is important to monitor the RNFL and the VD.

As mentioned above, there are many studies on the changes in RNFL and VD after silicone oil-filled vitrectomy for RRD, and most of the findings suggest that there is a negative effect on RNFL and VD after surgery, SB has a lower incidence of postoperative cataract and glaucoma than silicone oil-filled vitrectomy (22, 23), does not require vitrectomy, causes less damage, has a lower cost of surgery, and does not require prone position after surgery (24, 25). However, there is less research information on the effects of SB on peripapillary RNFL and VD production, so we objectively analysed the effects of SB on the retina by observing the changes in peripapillary RNFL thickness and VD in the preoperative and postoperative periods of RRD using OCTA. As there is still no objective criterion for the selection of surgical modalities for the treatment of RRD (26–28), it is also hoped that this study can provide an objective reference for better selection of surgical modalities.

Given the heterogeneity of RNFL thickness and VD distribution in different areas of the optic disc, pre-and post-operative changes may vary, in this study we divided the patients’ peripapillary retina into eight areas by OCTA and recorded and analysed the peripapillary RNFL and VD in detail at preoperative and different postoperative time points.

Considering that the scan quality affects the scan data and that the distribution of RNFL thickness and VD in different regions of the optic disc is inhomogeneous and may vary preoperatively and postoperatively, we recorded and analysed in detail the scanning SSI, the peripapillary global and the RNFL and VD of the peripapillary global division of the optic disc into eight regions by the OCTA instrument in each OCTA.

Ozdek et al. (29) used a scanning laser polarimeter to assess the changes in RNFL thickness around the optic disc after successful SB. They did not perform a statistical study of preoperative RNFL and scan quality. We found that there was no significant difference between the statistical results of SSI between the two groups, and the RNFL of the operated eye group was larger than that of the healthy eye group, suggesting that retinal detachment makes the periphery of the disc RNFL to increase. It has been suggested that the thickening of the RNFL in patients with RRD may be related to oedema or Müller cell hypertrophy and proliferation (7, 30). There was a significant decrease in SSI in the operated eye group within 2 weeks postoperatively, which could be due to a decrease in tear film quality or an increase in the postoperative inflammatory response, and as we could not exclude the effect of SSI on the RNFL scan at 2 weeks postoperatively, we could not differentiate whether the difference in peripapillary RNFL and VD between the two disc groups at 2 weeks postoperatively was due to the quality of the scans or due to the surgery, which could not be accurately assessed at this time. There was no significant difference in SSI between the two groups at 1 month postoperatively, and the areas that progressed further into the operated eye group over time were not significantly different from the healthy eye group. At 6 months post-operatively, there were no significant differences between the operated eye groups, except for two regions, tempo superior and tempo inferior, which remained different in the operated eye group compared to the healthy eye group. Which may indicate that most of the regions around the optic disc of the operated eye gradually approached and converged to the healthy eye after SB. Therefore, we believe that SB can improve the thickness of the RNFL, which is conducive to the recovery of visual function, and the recovery of the tempo superior and tempo inferior regions is slower, and it will take more than 6 months to fully recover to the level of the healthy eye.

It has been shown that RRD have higher retinal blood flow and better postoperative visual acuity (31). To investigate the effect of SB on retinal blood flow, an early study by Yoshida et al. (32) found that SB leads to a decrease in ocular blood flow perfusion, probably due to an increase in vascular resistance caused by pressurised objects, However, they mainly studied choroidal blood flow and only compared the postoperative period with the contralateral healthy eye, and did not monitor preoperative blood flow for comparison, which cannot exclude the effect of RRD itself on blood flow. Fineman et al. (33)reported a case of transient vision loss in a patient with scleral ring ligation and concluded that there is a significant reduction in ocular perfusion after ring ligation, leading to vision loss, but that this phenomenon is reversible. As SB surgery continues to evolve, clinicians tend to select segmental external compression and incise only the compressed quadrant of the conjunctiva to minimise damage, and the incidence of postoperative transient vision loss is relatively low.

There are a large number of findings on the changes in macular VD before and after SB in RRD: Bartolomé-Sesé et al. (34) investigated the changes in macular VD after vitrectomy combined with or without SB and SF6 as tamponade for the treatment of RRD, and the results of the study showed no significant changes in macular VD. Fallico et al. (35) investigated the changes in macular VD after SB in patients with RRD and showed that RRD causes a decrease in macular VD, but a trend towards an increase in VD was found at the 6-month postoperative follow-up. Barca et al. (36) found that regardless of whether RRD involved the macular region or not, the preoperative VD in the macular region of the operated eyes was lower than that of the healthy eyes, and after treatment with SB, there was a gradual recovery of the postoperative VD in the macular region. There was no significant difference from the healthy eyes at 6 months.

Results of studies on peripapillary VD are still scarce. Zabel et al. (6) analysed the retinal condition of 20 patients with retinal detachment. They performed fundus OCTA 6–14 months postoperatively and found that the VD of the operated eye was smaller than that of the contralateral healthy eye, but they did not perform preoperative OCTA on the patients and were unable to distinguish whether the reduction in blood flow was due to RRD disease effects or to surgery. In contrast, Nagahara et al. (37) observed fundus blood flow in 12 patients with RRD at 1 week, 1 month and 3 months after surgery and concluded that SB had a negative effect on retinal blood flow only in the pressure area and had no effect on retinal blood flow in other pressure areas, including the fundus of the optic disc. We found that the trend of peripapillary VD was similar to the changes in macular VD studied by Barca et al. Comparative analysis of peripapillary VD in the operated eyes group and the healthy eyes group showed that preoperative VD in all areas of the peripapillary disc was small compared to that of the healthy eyes. Still, postoperative VD in the operated eyes gradually recovered and there was no significant difference from that of the healthy eyes at 6 months, except for the superior temporal. However, the global value of the two groups are not significantly different. This suggests that, similar to the RNFL described above, we thought that SSI might adversely affect the scanning results of VD, but there was no significant difference in SSI between the two groups preoperatively, while the blood flow density in the operated eye group was significantly smaller than that in the healthy eye group, suggesting that RRD also adversely affects VD, and SSI was one of the factors for the lower blood flow in the operated eye group compared to that in the healthy eye group at 1 day and 2 weeks after surgery, with no significant difference between the operated and healthy eye groups in SSI at 1 month after surgery, and VD was higher than that in the healthy eye group. At 1 month after surgery, the SSI of the operated eye group was not significantly different from that of the healthy eye group. The VD had a tendency to gradually increase compared to that of the preoperative eye and gradually recovered to near that of the healthy eye, while the changes in each region were different, which needed to be studied separately, and it would take more than 6 months for their regions to fully recover to the level of the healthy eye.

Our study has the following limitations: Refractive error affects measurement results, and although we included patients with refractive error less than 3.00D, we did not include data on specific refractive error and axial length in the analysis, which may have introduced some error into the results, and because of the decrease in scan quality within 2 weeks postoperatively due to surgery, we did not exclude errors in the results due to SSI at this time, so the accuracy of deriving postoperative RNFL and VD values within 2 weeks is not high. All the patients we included had intraocular pressure within the normal range during the follow-up period, so we neglected the effect of intraocular pressure on RNFL and VD. All patients included in the analysis had no lesions in the contralateral eyes during the follow-up period, so we used a comparison of the healthy eyes contralateral to the operated eyes to study the effect of SB on RNFL and VD and did not include the normal population for comparison. Because the sample size was not large enough, we did not perform a more detailed subgroup analysis of whether different locations of the cingulate would have different effects on RNFL and VD in different areas of the peripapillary disc, but we divided the peripapillary disc region into 8 areas for separate analyses and there was a tendency for each area to be progressively closer to the healthy eyes, which may show that RNFL and VD recovered progressively after SB surgery. We will continue to increase the sample size, extend the follow-up time, and perform more detailed grouping for further investigation in the future.

In conclusion, SB does not negatively affect the peripapillary RNFL and VD after treatment of RRD, and both RNFL and VD progressively approach those of the healthy eyes after surgery. And it takes more than 6 months to fully recover to the level of healthy eyes.

## Data Availability

The original contributions presented in the study are included in the article/supplementary material, further inquiries can be directed to the corresponding author.
